# Performance of an Automated Detection Algorithm to Assess Objective Pulsatile Tinnitus

**DOI:** 10.1097/AUD.0000000000001301

**Published:** 2022-11-15

**Authors:** Sander W.J. Ubbink, J. Marc C. van Dijk, Rutger Hofman, Pim van Dijk

**Affiliations:** 1 Department of Otorhinolaryngology/Head and Neck Surgery, University Medical Center Groningen, The Netherlands.; 2 Graduate School of Medical Sciences (Research School Behavioral Cognitive Neuroscience), University of Groningen, The Netherlands.; 3 Department of Neurosurgery, University Medical Center Groningen, The Netherlands.

**Keywords:** Auscultation, Detection algorithm, Interobserver reliability, Pulsatile tinnitus, Objective tinnitus

## Abstract

In this paper we describe an automated detection algorithm that objectively detects pulsatile tinnitus (PT) and investigate its performance.

**Design:**

Sound measurements were made with a sensitive microphone placed in the outer ear canal in 36 PT-patients referred to our tertiary clinic, along with a registration of the heart rate. A novel algorithm expressed the coherence between the recorded sound and heart rate as a pulsatility index. This index was determined for 6 octave bands of the recorded sound. We assessed the performance of the detection algorithm by comparing it with the judgement of 3 blinded observers.

**Results:**

The algorithm showed good agreement compared with the majority judgement of the blinded observers (ROC AUC 0.83). Interobserver reliability for detecting PT in sound recordings by the three blinded observers was substantial (Fleiss’s κ=0.64).

**Conclusions:**

The algorithm may be a reliable alternative to subjective assessments of in-canal sound measurements in PT-patients, thus providing clinicians with an objective measure to differentiate between subjective and objective pulsatile tinnitus.

## INTRODUCTION

Pulsatile tinnitus (PT) occurs in 4-10% of all patients with tinnitus. Its auditory perception is usually pulse-synchronous. It is assumed the pulsatile sound originates from turbulent, sometimes increased blood flow in vessels of the cranium, head and neck area, and thoracic cavity. These sounds are transmitted to the cochlea by vascular or osseous structures.

According to literature the success of identifying the cause of PT strongly varies, ranging from 44-91% (Madani and Connor 2009). The diverse underlying pathology of PT makes it challenging to appoint PT to a specific diagnosis. The diagnostic pathway in PT-patients consists of full history taking, otorhinolaryngeal physical examination, and audiological testing. This is followed by non-invasive imaging techniques (Duplex echography; CT or CT-angiography (CTA); MRI or MR-angiography (MRA)). In most cases, a diagnosis is confirmed using these techniques (Mattox and Hudgins 2008; Deuschl et al. 2015; Pegge et al. 2017). If no clear diagnosis is made by non-invasive imaging, digital subtraction angiography (DSA) should be considered to rule out the presence of a dural arteriovenous fistula (DAVF), a life-threatening condition (van Dijk et al 2002).

In objective PT, the pulsatile sound can be heard by an examiner. The incidence of objective PT varies between 6-42% and with an objective PT the chance of finding underlying pathology is high (Madani and Connor 2009). Based on characteristics of the objectified PT, choices can be made on the preferred initial imaging (Mattox and Hudgins 2008; Ahsan et al. 2015), and furthermore, it may prevent patients from unnecessary imaging (Ubbink et al. 2019). Therefore, differentiating between a subjective and objective PT is beneficial.

To objectify PT, most often a stethoscope is used to detect the presence of a bruit in the neck or peri-auricular area. Thus, the detection of objective tinnitus depends on subjective assessment by the observer; however, this has several drawbacks and limitations. Whether a sound is detected depends on the proper function of the ear of the observer. Furthermore, experience is needed to detect and determine abnormal sounds, leading to variation in interpretation between experts. Similar drawbacks also apply to subjective evaluation of sound from the heart and lungs. Therefore, automatic detection and classification algorithms are already common for diagnosing heart and lung diseases (Pramono et al. 2017; Dwivedi et al. 2019). Since the incidence of PT is much lower, it is difficult to obtain enough clinical experience to detect and classify PT with auscultation, supporting the need for an objective method for detection and classification of PT sounds.

Several studies report the use of a microphone to objectify PT. With this technique, a sensitive microphone is placed in the external auditory canal. This method is more sensitive than conventional auscultation (Sismanis and Butts 1994). Moreover, with analysis of recorded sounds, differences in acoustic features between different pathologies can be demonstrated (Song et al. 2016). The method can even be used to perform treatment outcome evaluation (Kim et al. 2016) and may prevent patients with PT from unnecessary diagnostic imaging (Ubbink et al. 2019).

In this paper, we present an algorithm for automated sound analysis of sound recordings and analyse its performance to objectively detect PT. Furthermore, we investigated interobserver variability in objectifying PT in sound recordings to obtain insight in the reliability of the current method to objectify PT. Because in a previous study the suggestion was found that sound measurements in a group of patients with PT eligible for DSA might prevent patients from the risks of DSA (Ubbink et al. 2019), we validated the use of our algorithm in this specific subgroup of PT patients.

## METHODS

To investigate the performance of the algorithm in objectifying the presence of a pulsatile sound in patients with PT, we compared it to the subjective assessment of three blinded observers.

### Sound and PPG recording

All sound measurements were collected from patients with PT referred to our tertiary clinic for a DSA to rule out the presence of a DAVF. All patients had previous non-invasive imaging lacking a clear diagnosis. Measurements were performed in a sound-isolated chamber. Patients were seated in an upright position (torso and head upright). A sensitive microphone (Model ER10B, Etymotic Research Inc, Elk Grove Village, IL, USA) was placed in the external ear canal with an eartip that sealed the ear canal. Measurements were performed consecutively in both ears, starting with the non-symptomatic side. The signal was amplified and filtered with a lowpass filter at 15 kHz (Standford Research System, model SR640). The microphone was calibrated with a 14-mm eartip in a 1/2’’ microphone adapter using a sound calibrator playing a tone of 1 kHz at 94 dB SPL (Bruel&Kjaer, Type 4231). During sound measurement, a photoplethysmogram (PPG) was recorded at a fingertip of the participant (Nellcor DS100A SPO2-fingerclip sensor). A PPG is an optical technique using infrared light to detect volume changes in peripheral circulation and is used to monitor heart rate. The sound and PPG signals were simultaneously recorded and sampled at 44.1 kHz with 24-bit resolution (MOTU 624, Cambridge, MA, USA).

The ethics board of the University Medical Center Groningen (UMCG) approved the study protocol. All patients provided written informed consent. The study followed the tenets of the Declaration of Helsinki.

### Observer variability

Three blinded observers (with a background in audiology) judged the sound recordings on the presence of pulsatile sound. They were instructed to evaluate a heartbeat rhythm and were notified not to confuse it with breathing. There was no limit to the number of times to listen to each recording and they could adjust volume-settings. Interobserver variability between observers was assessed with Fleiss kappa (Ranganathan et al. 2017).

### Detection Algorithm

#### Pre-processing

All sound recordings and analyses were performed using custom routines developed with Matlab software (MathWorks Inc., 2016a, Natick, MA, USA). To remove high-frequency noise from the PPG-signal, it was filtered with a 5-Hz lowpass filter. The systolic peak from each heartbeat in the PPG-signal was used as a reference point to segment the sound recording. For each segment, an RMS-value was determined in dB SPL. To remove segments from the sound file with artefacts, e.g., swallowing or coughing, segments with a RMS-value exceeding the median RMS-value of all segments were excluded. All subsequent computations were performed on the resulting artefact-free signal.

#### Pulsatile-Tinnitus-Coherence-Index (PTCI)

The goal of the analysis algorithm is to assess whether sound recorded from the ear canal contains a component that is synchronous to the heartbeat. Therefore, the algorithm computes a measure of coherence between the sound recording and PPG-signal. The microphone signal was bandpass filtered (5^th^-order Butterworth) in six octave frequency bands, centred at 125; 250; 500; 1000; 2000 and 4000 Hz. A Hilbert Transform was used to compute the envelope for each band, which were subsequently smoothed with a 5-Hz low pass filter. Next, the magnitude squared coherence spectrum between the PPG-signal and the envelopes of the filtered signals were determined, respectively (MATLAB routine mscohere, with a Hamming Window of six seconds and an overlap of three seconds). The magnitude squared coherence spectrum is the frequency domain analogue of the correlation coefficient. For each band, a Pulsatile-Tinnitus-Coherence-Index (PTCI) was determined, defined as the maximum coherence value at the heart rate. The heart rate was determined by the frequency of the predominant peak, within the frequency range 0.8-2 Hz, of the FFT of the PPG-signal. A high value of this coherence index indicates that a pulsatile component related to the heart rate is present in the sound recording.

### Diagnostic performance of acoustical detection algorithm

The performance of the algorithm to detect a pulsatile sound was evaluated with the measure PTCI_max_ as a predictor variable, defined as the maximum PTCI-value across the six frequency bands. We assessed receiver operating characteristics (ROC) for the algorithm on detecting a pulsatile event using Matlab (Cardillo 2008). In this scenario, the algorithm was compared to the judgement by the majority of the blinded observers.

## RESULTS

### General Characteristics

Between 2015 and 2021, 36 PT-patients underwent DSA to rule out a DAVF. The population included 25 females and 11 males with a mean age of 55 (S.D. 15) years.

### Sound analysis

**Fig. 1.**
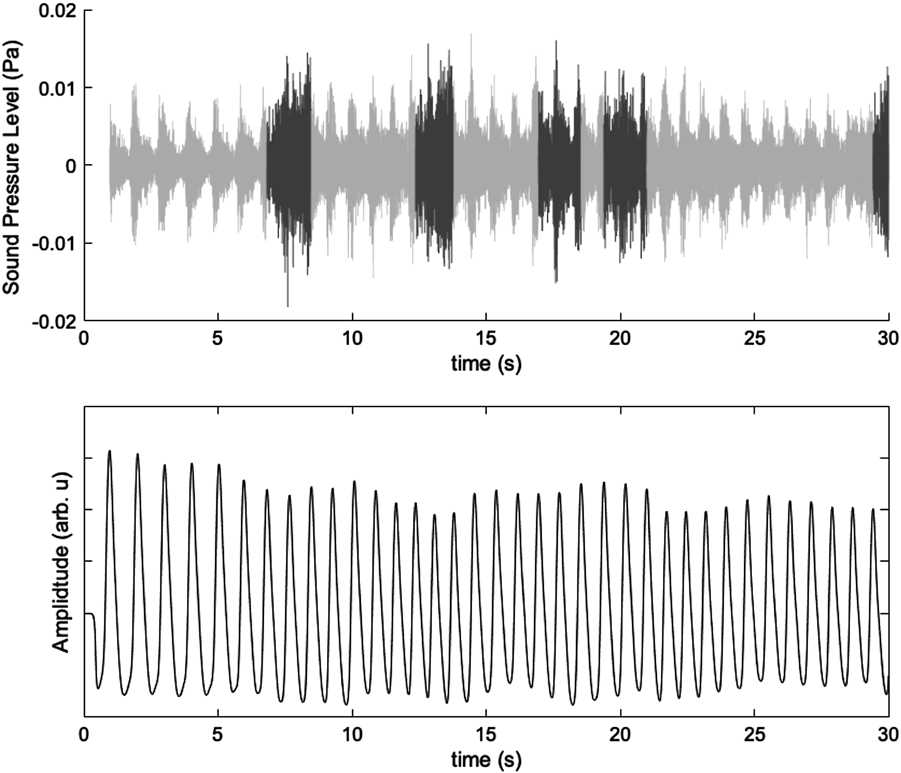
Sound (top) and PPG recording (bottom) of a patient with PT. The light-grey segments displayed were selected for further analysis. The dark-grey segments were removed after artefact rejection.

**Fig. 2.**
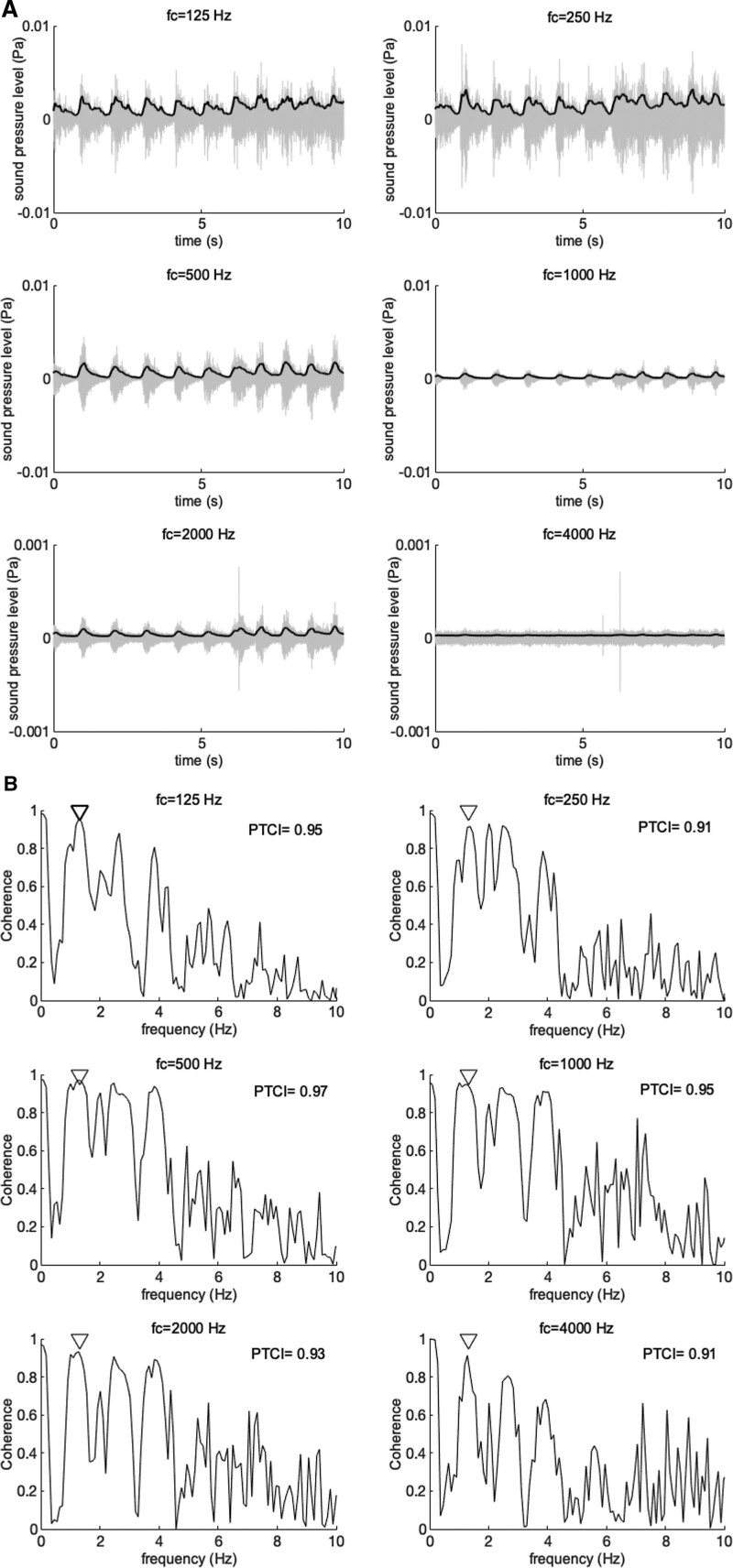
(A) A selection of 10 seconds of sound recording of a patient with PT divided into 6 frequency bands. The width of each band was one octave. Light-grey represents the filtered sounds. The black lines represent the Hilbert envelope, low-pass filtered with a cut-off frequency of 5 Hz. (B) Coherence spectrum of the bandpass-filtered sound and PPG-signal for the patient shown in panel A for frequency range 0-10 Hz. The heart rate frequency is indicated by a triangle. The PTCI value at the heart rate is displayed in the upper right corner.

Figure 1 shows an example of a sound and PPG recording obtained from a PT-patient. The segments from the sound recording that were determined as artefacts are shown in dark grey. In both the PPG recording and the sound recording a repeating pattern in the signal can be observed. Figure 2A shows a selection of 10 seconds of sound after band-pass filtering the selected segments of the sound recording in six octave bands. The envelope of the signal in each octave band shows a similar trend as the PPG recording. The coherence spectrum between the sound envelopes and the PPG signal for each octave band are displayed in Figure 2B. The spectra contain peaks at the heart rate frequency and its higher harmonic frequencies. The coherence index PTCI at the heart rate frequency for this PT-patient ranged from 0.91 to 0.97 across the six bands. The PTCI_max_ for the PT-patient in this case is 0.97; the PTCI value in the octave band with f_c_ 500 Hz.

### Interobserver variability

**Fig. 3.**
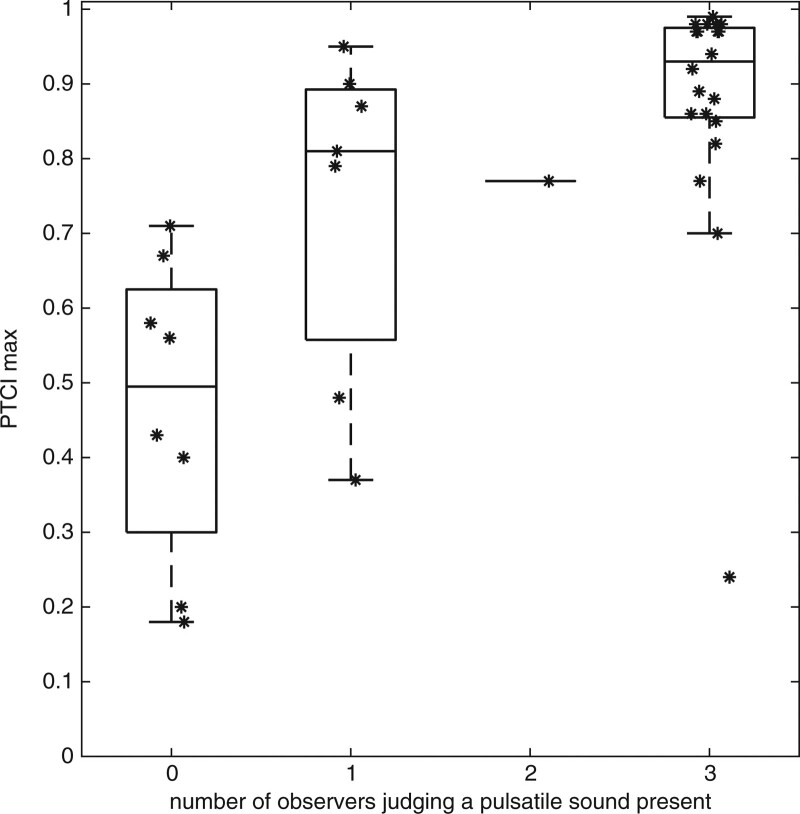
Combined boxplots and scatter plots showing PTCI_max_ (indicated with an asterisk) for all 36 sound recordings categorized on the number of observers that judged a pulsatile sound present in the sound recording. The top of the box represents the 75^th^ percentile, the bottom of the box represents the 25^th^ percentile, and the line in the middle represents the 50^th^ percentile. In the category with 2 observers judging a pulsatile sound present, there was only one sound recording. In this case only the 50^th^ percentile is indicated.

Figure 3A shows boxplots and scatterplots of PTCI_max_ as categorized on the number of observers that judged a pulsatile sound as present in the sound recording. In eight cases the observers did not agree. In seven of the eight cases, one observer that judged there was a pulsatile sound present. In one case, there were two observers that judged there was a pulsatile sound present. In 28 cases observers fully agree on the presence or absence of a pulsatile sound. The overall agreement (degree of concordance) was 83.33%. Fleiss’s kappa for the interobserver variability is 0.64 (95% confidence interval 0.43, 0.86). The agreement between observers in judging on the presence of PT in sound recordings is considered substantial (Landis and Koch 1977).

### Performance of sound algorithm

Figure 3 shows that sounds judged without pulsatility resulted in a low PTCI_max_ (median PTCI_max_ ~0.5; 25th and 75th percentile at ~0.3 and ~0.6). Sounds judged by all blinded observers with a pulsatility had a high PTCI_max_ (median PTCI_max_ ~ 0.93; 25th and 75th percentile at ~0.86 and ~0.98). This group had one outlier with PTCI_max_ 0.24. For sound recordings on which observers did not fully agree, PTCI_max_ values were between the aforementioned ranges. Based on the majority judgement of the observers, PT was considered present in 21 patients (≥2 observers judged a pulsatile sound present) and absent in 15 patients (≤1 observer judged a pulsatile sound present). ROC in which PTCI_max_ was used as a classifier to predict the outcome of the majority judgement of the observers resulted in an area under the curve (AUC) of 0.83, indicative of a good classifier performance. Table 1 shows some calculated sensitivity and specificity scores for several PTCI_max_ cut-off values. At a cut-off value of 0.7, sensitivity was 93% and specificity 69%.

**Table 1.** Several PTCI_max_ cut-off values and corresponding sensitivity and specificity scores for detecting a pulsatile sound.

PTCI_max_	Sensitivity (%)	Specificity (%)
0.6	97	54
0.7	93	69
0.8	80	81
0.9	53	88

## DISCUSSION

In this study, we presented an algorithm for automated detection of PT from sound measurements. The algorithm determines an index PTCI_max_ that can be used as an objective measure for the presence of a PT sound that is synchronous with the heart rate. It showed good agreement with the majority of subjective interpretations by the observers on the presence of a pulsatile sound (AUC 0.83).

Sound measurements to record PT have been presented earlier, but interpretation of these sound measurements was dependent on the judgement of the observer (Ubbink et al. 2019), interpretation of the observer on spectrogram (Song et al. 2016) or reporting on an overall sound pressure level (Kim et al. 2016). This is the first study reporting on a fully-automated algorithm-based sound analysis of PT which gives an objective measure on a continuous scale from 0 to 1 about the presence of a pulsatile sound per frequency band.

Furthermore, this study reports on interobserver reliability of judging the presence of PT. There was substantial agreement between observers in interpretation of sound recordings. Despite this substantial agreement, there are differences in the subjective interpretation between observers. This subjective interpretation also occurs when conventional auscultation is used to objectify PT. Presumably auscultation is less sensitive than in-canal sound measurements (Sismanis and Butts 1994). Furthermore, subjective assessments depend on the experience of the observers. These factors can lead to misclassification of PT as being subjective or objective. Proper classification of PT can determine the preferred choice of imaging (Shin et al. 2000; Sismanis 2001; Mattox and Hudgins 2008; Ahsan et al. 2015) and potentially prevent patients from unnecessary risk-full DSA imaging (Ubbink et al. 2019). The worst-case scenario would be missing a potentially lethal DAVF, such as when a clinician fails to identify a PT and decides not to perform DSA. This emphasises the necessity of an objective measure when differentiating in the presence of PT.

Our detection algorithm is based on a single audio feature, PTCI_max_. A detection algorithm with added audio features or additional pre-processing may improve accuracy, although this might also lead to overfitting. A next step would be to validate the performance of the current algorithm on more data, including a control group, or on other datasets.

The sound recordings judged by blinded observers with a pulsatile sound present had one obvious outlier. In this recording, the pulsatile sound was not continuously present above the microphone noise, which was noticed by the observers. The algorithm included segments with a pulsatile component present, as well as many segments with microphone noise, resulting in a PTCI_max_ of 0.24. By manual selection of segments in this sound recording, the PTCI value in multiple frequency bands increased above 0.8.

In six of the eight sounds on which the observers did not fully agree, the algorithm determined a PTCI_max_ value of 0.77 or higher, indicating that a pulsatile sound is probably present. Further analysis is needed to determine the difference between the algorithm and the interpretation of the observers. It is probable that the algorithm is more sensitive than the observers in detecting pulsatile components, since the pulsatile sound may have intensities beneath the hearing threshold of the observers or could be masked by sounds in other frequency regions. Another explanation might be that the blinded observers in this study did not have a consensus meeting. Also, a consensus meeting before (about criteria) or after judging the sounds (to achieve agreement) might have influenced interobserver variability and performance of our model.

In this study, the performance of the algorithm in objectifying PT was investigated. The diversity of aetiologies makes it challenging to assign PT to a specific diagnosis. It is known that frequency spectra of pulsatile sounds differ between aetiologies (Song et al. 2016). Our algorithm allows for this detection of pulsatile components in multiple frequency bands. A logical next step would be to investigate the clinical applicability of the algorithm in detecting a diagnosis, a specific pathology, and compare it to imaging currently used in the diagnostic follow-up of PT patients.

## CONCLUSION

The conventional method of objectifying PT by subjective assessments has limitations due to differences in interpretation by observers and the low incidence of PT. The automated detection algorithm in this study can serve as an objective measure to objectify PT. Additional research is warranted to determine the value of this objective measure in the diagnostic work-up of PT.

## Acknowledgments

S.U. performed experiments, analyzed data and wrote the paper; MvD, R.H. and PvD designed the experiment. All Authors discussed the results and implications and commented on the manuscript at all stages.
